# The Thirty-Third Annual Commencement of the Baltimore College of Dental Surgery

**Published:** 1873-03

**Authors:** 


### EDITORIAL, ETC.

*The Thirty-third Annual Commencement of the Baltimore College of Dental Surgery* was held at Concordia Opera House on Thursday evening, February 27th, 1873.

The main hall, aisles and galleries were so densely filled that many were unable to obtain even standing room inside. Before the exercises began, Prof. Winters' orchestra performed several airs in excellent style, which were highly appreciated by the brilliant assemblage. A large majority of those present were ladies, many of whom, being friends and acquaintances of the graduates, brought beautiful and costly floral remembrances, which, when arranged by the "bouquet committee," along the front of the stage, presented a beautiful appearance.

These floral testimonials were presented to the graduates immediately after receiving their diplomas; a basket being necessary to carry away the large number presented to one of the class, who resides in Baltimore, while others with difficulty held theirs with hands and arms, so popular were they with their lady friends.

Upon the stage were seated the Faculty of the College, the Graduating Class, Alumni, Professors of the various Medical Colleges of the city, and a number of other invited guests, among them Drs. W. W. H. Thackston, the only surviving member of the class of 1842, and W. H. Morgan, of the class of 1848, of Nashville, Tenn., late President of the American Dental Association.

The exercises commenced with an earnest and impressive prayer by the Rev. Dr. J. W. M. Williams, of the Central Baptist Church, who invoked the blessing of God upon the Faculty and members of the Graduating Class. The Dean of the College, Prof. Ferdinand J. S. Gorgas, then announced the names of the graduates, *twenty-seven* in number, the authority by which the degree of "Doctor of Dental Surgery" was conferred, and presented the following members of the Graduating Class with diplomas:

Andrew Abendschein, Maryland; Edward Alfred Becht, Holland; William Benson, Jr., Maryland; Joseph Clark Brown, North Carolina; Edward Wadsworth Bryan, Texas; George H. Chewning, Virginia; Eugene Crabbe, Virginia; J. Ormsby Donogh, Ohio; Miss Emile Foeking, Prussia; Solomon German, Maryland; Samuel Phillips Grafflin, Ohio; Charles Sylvester Grindall, Maryland; James A. Hart, Georgia; Samuel Thomas Henkle, M. D., Maryland; Frank Spencer Lewis, Maryland; Charles Wilhelm Lichtenberg, Illinois; James Smith Linthicum, M. D., Maryland; Calder Little, Maryland; Joseph McJordan, Virginia; Charles Hanson Morgan, W. Virginia; Robert Spotswood Switzer, Virginia; Jerome B. Ten Eyck, M. D., Michigan; John Hilery Vaughan, Virginia; John William Welsh, Maryland; George Milnor Wentworth, Massachusetts; George E. Wiley, Virginia; Jacob Henry Yost, Virginia.

After the conferring of the degrees, the Valedictory Address was delivered by Dr. W. W. H. Thackston, of Farmville, Virginia, in which he paid a high and truthful eulogium to the late Prof. Thomas E. Bond, M. D., one of the founders, and, up to his death, a Professor in the College. This address will appear in the next number of the *Journal.* The "Class Address" was then delivered by Jerome B. Ten Eyck, M. D., of the Graduating Class, in a manner which reflected credit upon himself, and also the class which he represented. The exercises closed with the benediction by the Rev. Dr. Williams, and the audience dispersed well pleased with the interesting features of the evening.

The Baltimore College of Dental Surgery having had the honor of conferring the first degree of "Doctor of Dental Surgery" in the world, has also graduated the first of the female sex who ever received a diploma in medicine or dentistry in Baltimore, in the person of Miss Emelie Foeking, of Prussia, who, after attending two full courses of lectures and demonstrations, passed a very creditable final examination. Miss F. conformed to all the rules and regulations of the College during the two sessions she was a student, no favor whatever as to requirement being asked for on her part, or extended to her by the Faculty on account of sex, and has fairly earned her degree by proficiency and earnest application. After a short time Miss F. will return to Berlin, where she intends to locate; and that she will succeed in establishing a large and lucrative practice, there can be no doubt, as she is well qualified professionally, and in manner so perfect a lady as to command the respect of all who know her.

The friends of the Baltimore College of Dental Surgery will rejoice in the prosperity of the institution, as evidenced by the session which has just closed; and the Faculty will do all in their power to sustain the high reputation of this pioneer institution of the world. The number of matriculants during the past session was 55. The thirty-fourth annual session will commence on the 15th of October, 1873, and close 1st March, 1874.

